# On Some Varieties of Paraplegia with Lateral Sclerosis, with Cases

**Published:** 1890-12

**Authors:** J. Michell Clarke

**Affiliations:** Assistant Physician and Pathologist, Bristol General Hospital; Assistant Lecturer on Physiology, Bristol Medical School


### ON SOME VARIETIES OF PARAPLEGIA WITH LATERAL SCLEROSIS, WITH CASES.

BY

J. MICHELL CLARKE, M.A., M.B. Camb., M.R.C.P. Lond.,

*Assistant Physician and Pathologist, Bristol General Hospital; Assistant Lecturer on Physiology, Bristol Medical School.*

---

I propose in this paper shortly to consider some cases of paraplegia in which the main symptoms were those that are taken to indicate sclerosis of the crossed pyramidal tracts in the spinal cord, and in which the arms were not affected.

The symptoms of this lateral sclerosis are paraplegia, with spasm and rigidity of muscles, and marked increase of myotatic irritability. The lateral sclerosis may arise in two chief ways: it may be the primary lesion, or it may be secondary (descending) to some other mischief in the cord. The symptoms resulting from it may exist unmixed, or be variously combined with concomitant symptoms due to disease of other parts of the cord, *e.g.* affections of sensation, ataxy of movement, atrophy of muscles, according as the lateral sclerosis is the sole lesion or is merely a part of the morbid process.

Adopting a pathological classification, in many ways the most convenient, we may divide the cases in which the chief or only symptoms of disease of the spinal cord are those due to lateral sclerosis, into two main groups: (I.) those in which the sclerosis, is primary in origin, and (II.) those in which it is secondary to some other lesion which interrupts the fibres of the crossed pyramidal tracts in some part of their course.

These groups may again be sub-divided into the following chief varieties:—

I. (1) Primary spastic paraplegia, primary lateral sclerosis, or spasmodic tabes dorsalis (Charcot), in which the lesion is probably confined to the lateral columns.

(2) Cases of general paralysis (of the insane) in which signs of lateral sclerosis come on early and attain severe degree, while mental symptoms are slight and unobtrusive.

(3) Ataxic paraplegia in which the lateral sclerosis is accompanied by degeneration in the posterior columns and of the direct lateral cerebellar tract.

II. In this group are included:

(1) Cases of "compression paraplegia," in which pressure is exerted upon the spinal cord—(*a*) by tubercular or malignant disease of the vertebræ; (*b*) by new growths in the meninges (fibrous or sarcomatous, syphilitic, inflammatory, hæmorrhagic, or tubercular); (*c*) as the result of injury to the spinal column.

(2) In certain cases of partial transverse (focal) myelitis in upper or mid-dorsal regions.

(3) In cases of disseminated sclerosis in which the sclerosed areas are so localised as to cause a descending lateral sclerosis without implication of other tracts or of the grey matter of the cord.

(4) Congenital form of spastic paraplegia of cerebral origin.

(5) In certain rare cases of central tumour of the cord in the lower cervical or upper dorsal region, and possibly in syringo-myelia, as in a case mentioned by Strümpell.

Lastly: cases of paraplegia in which there is no organic disease of the cord, but in which the symptoms of lateral sclerosis are simulated by certain functional conditions, must be placed in a category apart. In the first variety of Group I., the lesion is presumably confined to the fibres of the pyramidal tracts, including their terminal branches on their way to the motor cells of the anterior cornua through the ramifications of the grey matter. Hitherto there has, I believe, been no direct pathological evidence of degeneration accurately limited to these tracts, but on clinical grounds the occurrence of a pure lateral sclerosis is almost certain. Dr. Gowers is of opinion that the degeneration may sometimes be limited to the terminal branches of the pyramidal tracts in the grey matter, in which case sclerosis in the lateral columns would not be found *post mortem*. It is a "system" disease of the cord; that is to say, that fibres having a definite common function, and gathered together in a well-defined group or tract of constant position in the cord, are alone involved.

The following two cases under my care exhibit the typical features of this affection, with its slow onset, absence of evidence of any focal lesion, and of sensory symptoms. The first patient had had syphilis severely; in a certain number of cases syphilis appears to be the cause of the degeneration. In the other patient, the first signs of spinal-cord disease came on after typhoid fever; it is rare to find spastic paraplegia dating its origin from acute disease, and it is interesting to connote with this the condition of increased myotatic irritability without rigidity often present after one of the acute fevers or other severe wasting disease.

Edward S., æt. 26, labourer. His mother died of phthisis. He has had very heavy work as a labourer in the docks; has been accustomed to drink freely, and had syphilis five or six years ago. Eighteen months ago he had an attack, in which he thinks he was unconscious for a moment, but did not fall down. Since then he has noticed a very gradually increasing weakness and stiffness in both legs, but thinks that it began in the right. He suffers from a dull aching pain in the legs, and from cramping pains in the calves, with startings of the legs at night. There is obstinate constipation, but no bladder trouble. There has never been any affection of sensation. At the present time he is very weak, and trembles all over at the least excitement. He walks with both legs stiff and rigid, and moves them from the hips, without bending the knees; the toes scrape the ground as he walks, and the legs have a tendency to cross each other; he turns round with difficulty, and can hardly walk at all downhill. The muscles are well nourished and of good size; they react briskly to the Faradic and constant currents. There is great rigidity of the leg-muscles—clasp-knife rigidity; tickling the sole throws the whole leg into convulsions; there is knee- and ankle-clonus, and increased contraction to percussion of muscles themselves. Flexion and extension at knee and thigh decidedly weak. There is a little rigidity of movement about the right arm, and the tendon-reflexes are more active in the right than in the left arm. Otherwise the arms are unaffected.

All superficial reflexes present; no sensory affection; special senses normal; no loss of muscular sense, nor any ataxy of movement.

The second case was that of a charwoman, æt. 55, who had always had to work very hard. There was no history of alcohol or of syphilis. Seven years previously she had typhoid fever, and for some time afterwards suffered from pains in the back and weakness. About five years ago a gradually increasing weakness in the legs came on, and after a time compelled her to give up work. She has had some aching pains in the back, but none in the legs. The legs "start" at night. Walking is difficult; and if she knocks her legs together in walking, she falls. On examination, she is anæmic and badly nourished. She walks very stiffly and with manifest difficulty; her toes do not leave the ground, but scrape along the floor; she is unable to put the heels to the ground at the same time as the toes, on account of contraction in the calf-muscles. She can only turn round with great difficulty. There is no ataxy of movement, and she stands steadily with eyes closed. The muscles of the legs are very rigid, especially the flexors and extensors of the knee; the calf-muscles are rigid when she stands, but when she is lying down rigidity is less marked. The knee-jerks are much exaggerated, and there is slight ankle-clonus; front-tap contraction well marked; superficial reflexes normal. There is no muscular atrophy, and no affection of sensation; the muscles react well to the Faradic current. There is slight tenderness over the upper part of the sacrum and fifth lumbar spine, but no sign of any disease here. The bladder and rectum have been throughout quite unaffected. All arm movements are performed normally and with good strength. There is no affection of the special senses, nor of any cranial nerves. A few small patches of psoriasis are seen on the elbows, knees, and trunk. This patient has been treated by suspension three times a week for some three months, and is certainly better. Rigidity is less; she can walk better, though she still scrapes her toes; she is much stronger on her legs and can walk a great deal further. At the same time she has had the benefits of rest and good food whilst in the hospital, so that it is doubtful as to how much of the improvement can be attributed to suspension.

In certain cases of general paralysis (of the insane), the spinal symptoms predominate in the early stages, the mental symptoms are slight and indefinite, other of the more common signs are absent, and it may be doubtful as to whether we have to deal with a case of spinal cord disease, accompanied by slight mental failure, or with an irregular case of general paralysis. These cases are more likely to occur in ordinary hospital or private practice, and it is most important that the diagnosis should be made as early as possible, as it is only in the early stages of general paralysis that we can hope to check the disease. The spinal symptoms may indicate either that the posterior (type of locomotor ataxy) or that the lateral columns are affected: of the latter type, the two following cases are well-marked instances.

Albert C., a fitter, æt. 31 (in 1886). The patient's father was a heavy drinker, and a very nervous, excitable man, who could not bear the least noise when at home. The patient suffered from nocturnal incontinence of urine until ten years of age, and had a discharge from the left ear for some time. He had had rheumatism, but no syphilis or other illness.

I first saw him in October, 1886: he then complained of pains like cramp and stiffness in the legs, that he seemed to have no power in his legs, and at times could hardly drag himself along. His legs were drawn up when in bed; he complained of pains in head and temples, and that he could not bear any noise. His legs easily

"went to sleep" after sitting for some time. He walked stiffly, with slight scraping of his toes along the ground. There was rigidity, decided loss of power in the leg-muscles, with ankle-clonus and exaggerated knee-jerks on both sides; the superficial reflexes were brisk; muscular irritability to percussion (in legs). No affection of sensation; micturition and defæcation normally performed; pupils equal, acted well to light and accommodation. He was rather deaf; the other special senses were unaffected. These were the only symptoms at this time.

In December of the same year he had 24 "fits," in which there was momentary loss of consciousness; and for a short time after each fit he was unable to speak or to walk, and said that his limbs felt numb. After these fits he was much weaker; and his speech was not so distinct as before, showing a tendency to the blurring over of long words. His memory was bad, but he showed no other sign of mental failure; his friends stated, however, that he was apt to be excitable and childish at times, and had changed in disposition, showing no affection for his wife and family. He improved somewhat up till May, 1887, when he had another fit. The right pupil was now larger than the left; there was occasional nystagmus in lateral movements of eyes, and slight trembling of the lips.

After this, increase of paraplegia prevented further attendance at the hospital; he was unable to walk, but remained in much the same state mentally until December, 1887, when, after a severe fright, he became quite childish, and had delusions of grandeur. He was removed to an asylum, and after a time allowed to return to his friends. I saw him again in October, 1889: he was confined to his bed, was demented, could only utter meaningless sounds, had great difficulty in swallowing, and loss of control over bladder and rectum. There was general muscular rigidity of arms and legs, with contractures.

In the following case the progress of the disease has been slower, but I have no doubt that it is one of the same nature as the foregoing.

John Q., æt. 34, a shopman, came unner my care in August, 1886. He had had syphilis; diphtheria in November, 1885; but no other illness. He complained of weakness in the back and legs, gradually increasing during the last four months. He was giddy, had difficulty in turning round, and the legs trembled after any exertion. He suffered from morning sickness, but denied any alcoholic excess. His memory was bad; he walked stiffly and somewhat ataxically; there was weakness and slight rigidity of the leg-muscles; exaggerated knee-jerks, and slight but well-marked ankle-clonus. Sensation quite normal. The pupils were large but equal, and the right optic disc pale and somewhat atrophied.

He came again in August, 1888, stating that he had remained in about the same state till a month ago, when he had a slight apoplectiform seizure, followed by paresis of the left arm and leg, which subsequently passed away. His skin was greasy; his speech slurred and indistinct. His gait stiff, uncertain, and of the spasmodic character described by Dr. Savage, the feet when advanced and placed on the ground appearing to have a tendency to jump up. The other symptoms were unaltered.

In December, 1889, the weakness and rigidity of leg-muscles were decidedly worse, and ankle-clonus more pronounced. The speech was more slurred, there was fibrillar trembling of the tongue, and the lips trembled slightly when he spoke. Pupils equal; acted well to light. He complained much of stupidity in the head, and of inability to think or do continuous brain-work. He was certainly more feeble mentally, and his memory was very defective—so much so, that he forgot on one occasion that he had been at the hospital on the previous day.

In Ataxic paraplegia, the third variety of this group, besides sclerosis of the pyramidal tracts, there is degeneration of the posterior columns, and generally of the direct lateral cerebellar tract. In the posterior columns the sclerosis affects the postero-external column (c. of Burdach) to a varying extent in the sacral lumbar or lower dorsal regions, and extending from this there is degeneration of the columns of Goll. The distribution of the lesions marks the disease as a system disease. In addition to symptoms of lateral sclerosis, there is ataxy of movement from defect of co-ordination, due to the sclerosis of the ascending fibres from the posterior columns. The escape of the posterior root-zone accounts for the absence of sensory symptoms. In the progress of the disease the symptoms of lateral sclerosis tend to predominate, and the case may then closely resemble one of spastic paraplegia. The following case illustrates well the leading features of this variety.

The patient was a farm labourer of 35: he had had syphilis ten years ago, but no other illness, and had always been temperate. He complained of pain across the lower part of the back, and of weakness and stiffness in the legs. He could not stoop, and he felt that his legs trembled under him. There was loss of sexual power, and obstinate constipation; there was no urinary incontinence, but he was obliged to pass water immediately he felt that the bladder was full. The symptoms had come on gradually during the last five years. He has had no bad pain throughout, nor any loss of feeling in the legs; at one time there was some tingling in the toes at night. He is giddy in the dark, and has fallen down on getting out of bed at night. He was a fat, pasty-faced man. His walk, unsteady, became decidedly ataxic if he closed his eyes. He could not stand steadily, and when he closed his eyes swayed to and fro and would have fallen. There was also, with closed eyes, some ataxy of movement in the arms. Pupils equal; act to light and accommodation; no myosis; optic discs normal. Speech normal. The muscles of the legs and thighs were weak and somewhat rigid: this rigidity was most marked in movements at the knee and ankle. The knee-jerks were very much exaggerated; there was ankle-clonus on the left, but none on the right side; front-tap contraction well marked. Plantar reflexes very brisk; cremasteric not obtained; upper abdominal present, lower doubtful. Sensation to touch, pain, and temperature, normal. No tenderness, deformity, or other abnormality about the spinal column.

The defect of co-ordination in walking, standing, and performing various actions constitutes the chief difference from primary lateral sclerosis; while the absence of sensory affections and the increased myotatic irritability, with more or less muscular rigidity, distinguish the disease from locomotor ataxy, more especially from those more rare cases of the latter in which loss of power in the legs comes on early.

To pass to the second group, in which lateral sclerosis is of secondary origin. In those cases of disseminated sclerosis in which the patches affect the lateral columns only, it is impossible to make an accurate diagnosis. Those instances, however, are rare in which, even if the more common symptoms of disseminated sclerosis—*e.g.* characteristic tremor, nystagmus, scanning speech—are absent, there are not other signs, such as localized areas of anæsthesia, paralysis or atrophy limited to a particular group of muscles, or to those supplied by one nerve, to indicate that the morbid process is not confined to one system of fibres. If the distinction cannot be made at once, the lapse of a little time generally clears up the case by the appearance of fresh symptoms.

Next to these cases, the greatest difficulty in diagnosis from primary spastic paraplegia is presented by cases of "compression" paraplegia in which the fact of compression cannot be determined, and the pressure exercised on the cord is slight and gradually produced. Under such circumstances no sensory disturbance may be excited, and paresis, with excessive myotatic irritability, may be the only symptoms. Patches of transverse myelitis in the upper dorsal region may also be for some time confined to the lateral columns and be symmetrical in distribution, so as to bring about a descending lateral sclerosis without any affection of sensation and without focal symptoms; as in the last instance, a paralysis of the legs of spastic type will be produced. Tumours in the lower cervical and upper dorsal cord are said rarely to give rise to the same symptoms; and in one case, as mentioned above, syringomyelia was the lesion found at the autopsy. In the distinction of syringomyelia, Charcot relies on the presence of a peculiar disorder of sensibility; namely, tactile sensation is normally preserved, while sensation to heat and cold and to pain is lost. In the foregoing statement I do not of course mean to imply that in most cases of myelitis or compression paraplegia, where these diseases produce their ordinary train of symptoms, there is any difficulty in the diagnosis from pure spastic paraplegia, but only that in certain cases the difficulty is a very real one. The following case illustrates the doubt there may be as to the exact diagnosis :

T.B., a coppersmith, æt. 31, comes of a healthy family, and had never had syphilis nor any bad illness, until in 1885 he was operated upon for a large growth in the left arm, which turned out to be a sarcoma. Following the operation, he suffered from abscesses of the elbow-joint and erysipelas, and was in bed altogether for four months. When he came to get about again he found that his legs were weak and stiff; and this weakness and stiffness has grown gradually worse, until now he can only walk with difficulty a short distance. Except for a dull aching pain in the back, he has been quite free from pain, and has had no loss of sensation. There has been a feeling of "heaviness" over the left leg, and the muscles twitch at night: the left leg was the first affected, and he feels the weakness most in the left knee. He suffers from constipation, but throughout has had complete control over the bladder and rectum. He has never had a girdle pain.

In walking the lower limbs are stiff and rigid, but when lying on the couch he moves his legs fairly well without ataxy. Except for the paraplegia, he is quite healthy. There is a slight lateral curvature of dorsal spine to the left, with curve to right in lumbar region: this he states has always existed. There is not the least prominence, pain, or tenderness over any of the vertebral spines. Sensation normal to pain, touch, and temperature, and equal on both sides on careful testing. The muscles are everywhere well nourished. The muscles of the legs are weak, especially at the knee; there is marked rigidity of the "clasp-knife" kind. Knee-clonus is well marked; ankle-clonus could not be obtained in the ordinary way, on account of active contracture of the muscles; but whilst turning over on the bed he rested his weight for a moment on the toes of the right side, and this started most violent prolonged clonic contractions of the whole limb. Plantar reflexes brisk; cremasteric, lower abdominal, and gluteal, absent; epigastric present. Arms unaffected; special senses normal; no cranial nerve paralysis. Although the symptoms closely correspond to it, I do not think that we have here to do with primary spastic paralysis. The lateral curvature had always been present ever since the patient could remember, and had shown no increase of late years, and cannot therefore be held in any way accountable for the paraplegia. The rate of onset—distinct paralysis produced in four months—is very rapid for primary spastic paraplegia, and the absence of some of the superficial reflexes may be indicative of mischief in the lower dorsal and lumbar regions. The nature of the lesion remains uncertain: it is possibly a patch of myelitis, with secondary lateral sclerosis descending from it. The stationary character of the symptoms negatives the presence of a small tumour in the cord secondary to the sarcoma removed from the arm, unless this had for some reason ceased to grow. The patient's walking powers have improved decidedly under treatment by suspension: he considers that his legs are less stiff, and says that they do not "start" so much at night; but so far as I could ascertain, rigidity and increase of myotatic irritability remain about the same.

In the following case, the history given by the patient indicated that the lesion was most probably a limited patch of myelitis, perhaps induced by the fall on the back two months before: the symptoms when he came under observation were those of spastic paraplegia, due to the lateral sclerosis which followed, and were more marked on the right side than on the left. The patient, a healthy-looking man of 49 years of age, was a sailor. He had always been strong and temperate, and had not had syphilis. He had a fall on the back twelve months ago, of which he thought nothing at the time; but two months later he had an attack of pain in the lumbar spine during the night, and since then has been losing strength in the legs, in the right more than in the left, so that he cannot stand or walk as he used to. He has had no pain or other affection of sensation; no girdle pain; no difficulty in micturition or defæcation.

On examination, he complained that the right leg felt cold; but no objective difference could be made out in this respect between the two limbs, and sensation of all kinds was normal over both sides. He walked feebly, dragging the right leg, and, after walking a little time, scraped the toes along the ground. The muscles of the legs were rigid, especially on the right side: they were well nourished, but weak. Ankle-clonus was easily obtained; the knee-jerks exaggerated, the right most so, and plantar reflex was more brisk on this side. Except the right cremasteric, which was absent, the other superficial reflexes were normal. No loss of muscular sense; electrical reactions normal; arms unaffected; no abnormality detected in spinal column, and no pain nor tenderness in any spot.

There are two chief "functional" conditions in which the symptoms resemble those of spastic paraplegia, but in which there is probably no structural change in the cord,—at any rate, sufficiently coarse to be recognisable by the microscope.

The cases which bear the closest resemblance to lateral sclerosis are those to which Dr. Hughes Bennett gave the name of "hypertonic paralysis."* They are characterised by motor weakness, with excessive myotatic irritability, but without rigidity. Every degree of increased muscular irritability to percussion on the mass of the muscles themselves or on their tendons may be present. Although the condition is a general one, and the whole muscular system is affected, weakness of the lower extremities often predominates. The chief symptom complained of by the patient is then a gradually increasing weakness in the legs, first shown by the readiness with which fatigue is induced after slight exertion. There is no affection of sensation, nor of the special senses, nor of the bladder or rectum; the muscles do not atrophy, but in protracted cases the affected limbs may become smaller from disuse. The weakness may come on after prolonged severe exertion: it may quickly pass away again, may last a considerable time with gradual improvement, or may remain stationary. I do not think that these cases can be separated from the very similar condition of weakness with excessive muscular irritability to mechanical stimulation which is met with in wasting diseases such as phthisis, or in convalescence from the acute fevers, except that in the latter the muscular wasting from malnutrition is more marked. In rheumatoid arthritis a similar condition of the muscular system obtains in its most extreme form, with a very much greater degree of wasting. The electrical reactions are normal or show slight increase to both currents.

* Brain, January, 1888.

Clinically, the cases of which Dr. Bennett wrote are distinguished by the occurrence of this "hypertonic paralysis," without as a rule obvious exciting cause and unattended generally by wasting of the muscles, although these may be small and flabby. The following case illustrates the typical features of this affection, and it is needless to quote the details of other cases of which I have notes. My patients have been chiefly men, in early adult life; as a rule there is not an obvious exciting cause, but the patients were generally engaged in some exhausting and laborious occupation, carried physical exercises to an extreme degree, or gave way to excesses of various kinds:

W. S., æt. 18, a blacksmith, of healthy family, had had no previous illness until twelve weeks previously, when he had a bad cold. He remained at work, feeling weak, until fourteen days before he came to me (in March, 1889); on this day his legs seemed to give way under him whilst he was at work—he fell down, and was unable to walk home: at the same time he had a pain in the right knee. Since then he has been unable to stand or walk for long at a time. For some weeks before the fall he had felt a gradually increasing weakness in the legs. He had not had any pain, nor other sensory disturbance. On examination he appeared healthy, except that he was rather anæmic; the thoracic and abdominal organs were sound. The muscles were weak and rather flabby, but showed no wasting nor rigidity; movements were weak, but normally performed; his gait shuffling and rather unsteady. Knee-jerks much exaggerated; ankle-clonus on both sides—most on right. The tendon reflexes in the arms also showed marked increase, and the muscles generally gave an exaggerated response on percussing them. Plantar reflexes absent; other superficial reflexes normal. On examining eyes fundus and optic discs healthy. $V=\frac{20}{30}$. He was kept at rest on full diet, and given a ferruginous mixture containing arsenic and belladonna; and made a good recovery in about eight weeks, at the end of which time ankle-clonus could no longer be obtained, while the knee-jerks were still somewhat exaggerated, but subsequently gradually became normal.

These cases of "hypertonic paralysis" resemble mild cases of spastic paralysis, in an early period of the disease, but differ from it in the absence of muscular rigidity, and in the increase of myotatic irritability affecting the muscular system generally, arms as well as legs; Ankle-clonus may be pronounced; in two instances I found that it was absent so long as the patient remained in bed, but if he were allowed to get up and walk about the room a little, it could be elicited in the most marked way. This very distinct increase of clonus on exertion may be found useful in the diagnosis from spastic paraplegia, in which the same degree of difference is not found. Dr. Bennett lays stress on the frequent absence of the plantar reflex, as in cases of hysterical paraplegia. He states that, so far as is at present known, these cases either recover or remain in a stationary condition, and do not go on to spastic paraplegia with lateral sclerosis. I have given above, however,—and others are on record,—a case in which spastic paraplegia distinctly came on after enteric fever, though it took five years in developing to complete paralysis; and as this increased muscular tone or irritability with motor weakness is a common sequela of acute fevers, it would seem *a priori* probable that some cases do not entirely recover, but eventually pass by slow intermediate stages into well-defined lateral sclerosis. Lastly: cases of so-called hysterical paraplegia may present rigidity of leg-muscles, with increase of myotatic irritability, without any affection of sensation. Aid in diagnosis may be obtained from the general bearing of the patient, the account of the present illness and of its cause, while the history may disclose previous attacks of recognized hysterical affections. Other symptoms, such as marked ovarian hyperæsthesia, may be present. Of the local signs, whilst the knee-jerk may be exaggerated and there may be ankle-clonus, this latter is always slight and badly marked, and never present in the extreme degree common to spastic paraplegia. The plantar reflex, as Buzzard points out, is generally absent, while the other superficial reflexes are natural. Tapping the patellar tendon may evoke a nervous start or a display of nervous excitement; rigidity is present in all positions of the limb, and the resistance to passive movement may be felt to vary from time to time. A contracture of the great toes in the position of extension is a not uncommon characteristic.

